# H1N1 influenza viruses varying widely in hemagglutinin stability transmit efficiently from swine to swine and to ferrets

**DOI:** 10.1371/journal.ppat.1006276

**Published:** 2017-03-10

**Authors:** Marion Russier, Guohua Yang, Atanaska Marinova-Petkova, Peter Vogel, Bryan S. Kaplan, Richard J. Webby, Charles J. Russell

**Affiliations:** 1 Department of Infectious Diseases, St. Jude Children’s Research Hospital, Memphis, Tennessee, United States; 2 Department of Pathology, St. Jude Children’s Research Hospital, Memphis, Tennessee, United States; 3 Department of Microbiology, Immunology & Biochemistry, College of Medicine, University of Tennessee Health Science Center, Memphis, Tennessee, United States; Emory University School of Medicine, UNITED STATES

## Abstract

A pandemic-capable influenza virus requires a hemagglutinin (HA) surface glycoprotein that is immunologically unseen by most people and is capable of supporting replication and transmission in humans. HA stabilization has been linked to 2009 pH1N1 pandemic potential in humans and H5N1 airborne transmissibility in the ferret model. Swine have served as an intermediate host for zoonotic influenza viruses, yet the evolutionary pressure exerted by this host on HA stability was unknown. For over 70 contemporary swine H1 and H3 isolates, we measured HA activation pH to range from pH 5.1 to 5.9 for H1 viruses and pH 5.3 to 5.8 for H3 viruses. Thus, contemporary swine isolates vary widely in HA stability, having values favored by both avian (pH >5.5) and human and ferret (pH ≤5.5) species. Using an early 2009 pandemic H1N1 (pH1N1) virus backbone, we generated three viruses differing by one HA residue that only altered HA stability: WT (pH 5.5), HA1-Y17H (pH 6.0), and HA2-R106K (pH 5.3). All three replicated in pigs and transmitted from pig-to-pig and pig-to-ferret. WT and R106 viruses maintained HA genotype and phenotype after transmission. Y17H (pH 6.0) acquired HA mutations that stabilized the HA protein to pH 5.8 after transmission to pigs and 5.5 after transmission to ferrets. Overall, we found swine support a broad range of HA activation pH for contact transmission and many recent swine H1N1 and H3N2 isolates have stabilized (human-like) HA proteins. This constitutes a heightened pandemic risk and underscores the importance of ongoing surveillance and control efforts for swine viruses.

## Author summary

Many genetically diverse influenza viruses circulate among wild aquatic birds. Occasionally, one causes an outbreak in domestic poultry, swine, other animals, or humans. Zoonotic influenza viruses rarely cause a human pandemic. The hemagglutinin (HA) surface glycoprotein is the major surface antigen. A pandemic-capable virus requires an HA protein immunologically unseen by most people. During entry, HA binds sialic-acid-terminating receptors. A switch of preference from avian- to human-preferred receptor form is necessary but not sufficient for pandemic potential. What else is needed? After receptor binding, the virus is endocytosed. Acidification triggers the HA protein to undergo irreversible structural changes that cause membrane fusion, allowing genome delivery. Avian viruses tend to have unstable (easy to activate) HA proteins while humanized viruses prefer greater stability. Here, we show pigs permit replication and contact transmission of both stable and unstable HA proteins to recipient pigs. Thus, swine may accommodate both human-like and avian-like HA stability and receptor-binding properties, which have been linked to pandemic potential. Many swine viruses isolated since 2009 contain HA proteins already humanized with respect to receptor binding and stability. One could cause the next pandemic if it becomes immunologically distinct from human influenza viruses.

## Introduction

Numerous influenza A viruses (IAVs) exhibiting great diversity circulate in various host species, yet few strains evolve the traits necessary to jump between species and sustain an epidemic. The largest pool of IAVs is maintained in a global reservoir of wild aquatic birds [1]. Between them, avian IAVs include 16 hemagglutinin (HA) and 9 neuraminidase (NA) antigenic subtypes in many combinations [2,3], which are designated H1-H16 and N1-N9, respectively. The H17N10 and H18N11 subtypes have recently been discovered in bats [4,5], although the extent to which these viruses are capable of transmission to other species is not yet known. Over the past century, a few avian IAVs have been able to establish long-term epidemics in domestic poultry and swine with human infection resulting from close contact with infected animals [6]. Avian H5, H7, and H9 subtypes caused outbreaks in poultry and have resulted in spillover into humans with limited human-to-human transmission. Domestic swine have proven highly capable of serving as bridging hosts for the adaptation of avian IAVs to replication in humans [1]. Swine are susceptible to many avian and human strains and can serve as a mixing vessel for the reassortment of 8 gene segments from different IAVs [7]. The current IAV epidemics in domestic swine herds are caused by H1N1, H1N2, and H3N2 strains. IAV diversity in swine is increased by antigenic drift [8,9]. In 2009, a swine H1N1 virus emerged in humans and rapidly spread globally, causing a pandemic within months [10,11]. The 2009 pandemic H1N1 (pH1N1) virus is the result of reassortment between viruses containing genes from classical swine IAVs (HA gene), Eurasian swine IAVs (NA and M genes), and triple-reassortant swine IAVs that contain internal genes derived from swine, human, and avian influenza viruses (the M, NS, and NP genes are derived from classical North American swine IAV, the PB1 gene from a human IAV, and the PB2 gene from an avian IAV) [11]. This complex reassortment of pH1N1 in pigs suggests that an optimized gene constellation helps promote the emergence of a human pandemic virus.

Two well-established properties linked to IAV interspecies adaptation include polymerase activity and receptor binding [12]. Avian and human IAVs replicate efficiently at approximately 41°C and 33°C, respectively [13]. These temperatures correspond, respectively, to those of the avian enteric and human upper respiratory tracts. Mammalian adaptation has been linked to mutations in the PB2 protein at positions 591, 627, and 701 that increase polymerase activity and replication at the lower temperature [13–16]. During IAV entry, the HA protein binds sialic acid–containing receptors on the cell surface, the virus is internalized by endocytosis, and the HA protein is triggered by acidic pH to undergo irreversible structural changes that cause membrane fusion and release of the viral genome into the host cell cytosol [17]. Human- and avian-adapted IAVs bind preferentially to α-2,6- and α-2,3-linked sialic acid receptors, respectively, which are differentially expressed in various hosts, cells, and tissues [18–22]. Accordingly, human influenza viruses have shown a tendency to bind more strongly to α-2,6-containing non-ciliated cells in mammalian trachea and bronchi as well as type I pneumocytes in the lungs; whereas, avian influenza viruses appear to have a preference for binding α-2,3-containing ciliated cells and type II pneumocytes [18,19]. Both α-2,6- and α-2,3-linked sialic acid receptors have been extensively detected in pigs, having a respiratory distribution similar to that observed in humans [20]. Adaptation of IAVs to mammalian receptor binding has been associated with mutations in the receptor-binding pocket that switch receptor specificity to α-2,6 at HA positions 190, 225, 226, and 228 (H3 numbering) [23–29]. Swine express both α-2,3- and α-2,6 forms of sialic acid receptors, permitting entry by IAVs with either receptor specificity [20,30,31] and thereby facilitating their role as bridging hosts.

A third trait linked to interspecies adaptation of IAVs is HA acid stability, which is commonly defined as the activation pH at which irreversible HA conformational changes are triggered [32,33]. Avian influenza viruses tend to have relatively unstable HA proteins that are triggered at a higher activation pH than are those of human- and ferret-adapted IAVs [34–41]. This may not be universally true, as six H1N1 IAVs isolated from ducks and coots between 1976 and 1980 have HA activation pH values similar to those of human seasonal IAVs [42]. For H5N1, a relatively unstable HA (activation pH 5.6–6.0) is necessary for efficient replication and transmissibility in avian hosts [34,38]. In ferrets, a more stable HA protein (activation pH < 5.6) is needed for efficient upper respiratory tract growth and airborne transmissibility of H5N1 [36,37,43,44] and pH1N1 viruses [39]. Furthermore, a stable HA protein has also been linked to pH1N1 pandemic potential and adaptation to humans [39,45].

The permissible range of HA activation pH in pigs and the pathways available for interspecies adaptation of this property may depend in part on the virus genetic constellation. Swine influenza viruses are highly diverse and have a complex evolutionary history in North America and Eurasia [46,47]. H1N1, H1N2, and H3N2 subtypes are currently endemic in pigs [48]. With respect to H1 viruses, the classical lineage was first isolated in North America in 1930 [49], before which the clinical symptoms of influenza in pigs were described during the 1918 Spanish influenza pandemic [50]. The classical lineage (H1α cluster) remained dominant until the emergence of triple-reassortant swine viruses around 1998 [46], after which there was a dramatic increase in swine influenza virus diversity [8]. H1β swine viruses were first detected in 2001–2002, H1δ (or “seasonal human-like” swine H1) in 2003–2005, and H1γ strains in 1999–2000 [51].

Despite recognition that HA stabilization may be necessary for the adaptation of avian-like IAVs to ferrets and humans, the role of swine in this process is unknown. Pre-2009 swine H1 virus isolates have HA activation pH values ranging from pH 5.4 to 6.0, early human 2009 pH1N1 isolates have HA activation pH values of 5.5 to 5.6, and subsequent human-adapted 2010–2012 human isolates range from 5.2 to 5.4 [39,42]. These observations suggest an unstable HA may become stabilized to intermediate stability in pigs before the virus jumps to humans and HA becomes further stabilized. Other pathways may be possible, yet have not been investigated. To determine the importance of HA stability for replication in and transmission from swine, we measured the HA activation pH values of recently isolated H1 and H3 swine IAVs and evaluated experimentally the impact of HA activation pH on replication in swine, as well as on swine-to-swine and swine-to-ferret transmission. The results show swine permit replication and transmission by influenza viruses varying widely in acid stability.

## Results

### Contemporary swine IAVs vary widely in HA stability

**Fig 1:**
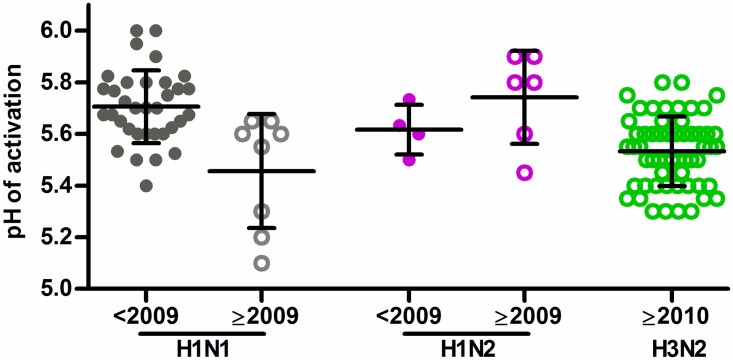
HA activation pH values for swine H1N1, H1N2, and H3N2 influenza viruses. Each data point represents the pH of activation of an individual virus, as measured by syncytium assay with 2 to 4 duplicates. Activation pH values of prepandemic (<2009) swine H1 viruses were previously reported [39] and are used here for comparison. A list of contemporary swine H1 and H3 viruses is given in S1 and S2 Tables.

We measured the HA activation pH for 14 swine H1N1 and H1N2 IAVs isolated in 2009–2016 (Fig 1) using a syncytia assay. These viruses contained HA genes of β, γ, δ1, γ-pandemic-like, and pandemic lineages [52]. One of the isolates with the least HA stability, sw/NE/4D-0114-P14/2014 (pH 5.9), contains a pandemic-lineage HA gene (S1 Table). This does not preclude swine from being infected with or transmitting influenza viruses containing HA proteins that are more acid stable. For example, the 2 contemporary swine H1 isolates with the greatest HA stability, sw/NE/4G-0314-P18/2014 (pH 5.1) and sw/GA/1E-0214-P26/2014 (pH 5.2), also contain HA genes from the human pandemic lineage. Overall, the contemporary swine H1 viruses had HA activation pH values ranging from 5.1 to 5.9 (S1 Table), which is a broader and significantly lower pH range (P < 0.05) than that of pre-2009 swine H1 isolates, for which the HA activation pH values ranged from 5.4 to 6.0 (Fig 1). We next measured the HA activation pH for 57 contemporary swine H3N2 IAVs (Fig 1). These viruses had HA activation pH values ranging from 5.3 to 5.8 (S2 Table), overlapping on the lower end with human seasonal and pandemic H3 IAVs [35]. Overall, the data show that swine support infection by contemporary H1 and H3 IAVs that have a broad range of HA stability and are in many cases acid stable (activation pH < 5.5).

### Infectivity in pigs by pH1N1 viruses that vary widely in HA stability

To determine experimentally the permissible range of HA activation pH for IAV replication and transmission in swine, we selected 3 previously characterized pH1N1 viruses that have a common A/TN/1-560/2009 (pH1N1) backbone: WT (pH 5.5), HA2-R106K (5.3), and HA1-Y17H (6.0) [39]. The two mutant viruses differ from the WT virus by a single amino-acid residue in their HA and have WT-like properties of expression, cleavage, and preferential α-2,6-linked sialic acid binding. All 3 viruses also had similar replication kinetics in MDCK, A549, and NHBE cells. Compared to WT and R106K, Y17H with a destabilized HA protein (pH 6.0) had reduced replication and was a loss-of-function mutant for airborne transmission in ferrets [39].

A/TN/1-560/2009 (pH1N1) was isolated during the initial stages of the 2009 pandemic; thus, its lineage demonstrated pandemic capability in humans [53]. On multiple occasions, starting in 2009, pH1N1 viruses were transmitted back to swine [54], showing that the lineage retained an ability to infect swine. A/TN/1-560/2009 and the related A/CA/04/2009 replicated efficiently in ferrets and could be transmitted to them by the airborne route [39,55]. Ferrets have airway characteristics similar to those of humans, and this animal model is widely accepted to be well suited for studying the pathogenicity and transmissibility of IAV as observed in humans [56]. Because A/TN/1-560/2009 (pH1N1) and closely related viruses replicate in and are transmitted in swine, ferrets, and humans, we considered this genetic backbone to be well suited for studying interspecies transmission.

**Fig 2:**
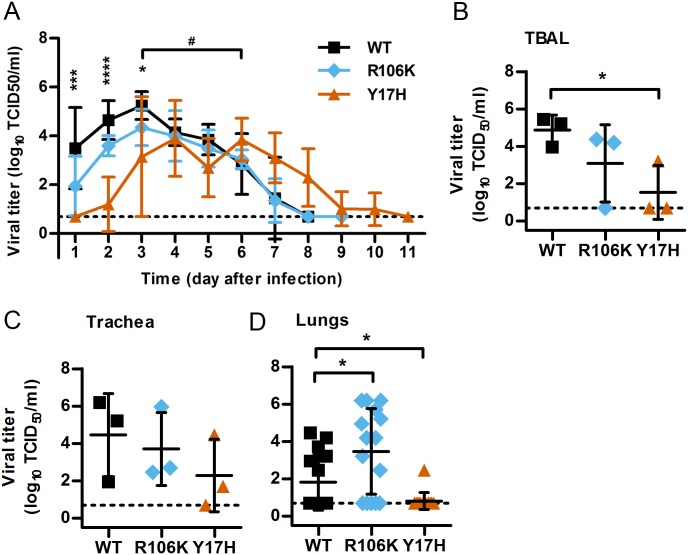
Viral growth in the upper and lower respiratory tract of pigs. Three-week-old piglets were inoculated intranasally with 1.4 × 10^6^ PFU of WT, Y17H, or R106K viruses in PBS. (A) Mean (± SD) virus titers in the nasal cavity of pigs (n = 5). Nasal swabs were collected and titrated by TCID50. Two-way ANOVA with Bonferroni multiple comparisons was performed, and significant differences between the WT and Y17H groups at time points (*) and at the peak of infection (^#^) are shown as follows:*^,#^*P* < 0.05, ***P < 0.001, ****P < 0.0001. There was no significant difference between the WT and R106K groups. (B, C, D) Mean (± SD) virus titers in the lower respiratory tract of pigs (n = 3). Animals were euthanized on day 3 after inoculation. Tracheobronchoalveolar lavage (TBAL) fluid (B) and tissues (C, D) were collected and titrated by TCID50. Comparisons between groups were performed using Student *t*-tests; *P < 0.05.

In 2 separate experiments, we intranasally inoculated a total of 8 pigs with pH1N1 WT, Y17H, or R106K viruses: 5 to be used for daily measurements of the viral load in nasal swabs and 3 in which to examine the tissue titers, histopathology, and inflammatory responses at 3 days post-inoculation (dpi). All of the pigs seroconverted, with no significant differences being observed between the virus groups in terms of serum antibody titers at 14 dpi (S1A Fig). Compared to WT virus, the stabilized R106K mutant displayed no statistically significant differences with respect to the viral load in nasal swabs, tracheal homogenates, or tracheobronchoalveolar lavage (TBAL) fluid (Fig 2). Histopathologic analyses also showed similar spread by WT and R106K viruses in the nasal turbinates and trachea (Table 1). In contrast, the R106K mutant showed greater spread in the lungs by immunohistochemistry, perhaps explaining in part why lung viral loads in the R106K group were an average of 30-fold higher (P < 0.05) than in the WT group (Fig 2D). Compared to WT virus, the destabilized Y17H mutant yielded peak nasal swab titers that were delayed by 3 days and reduced 30-fold (*P* < 0.05); Y17H viral loads were also significantly reduced in TBAL fluid and lung homogenates (Fig 2). Two of the three Y17H-infected pigs were negative for NP by immunohistochemical staining (IHC) in their nasal turbinates, tracheae, and lungs. In contrast, all tissues were positive in WT-infected piglets except for the trachea of 1 animal (Table 1).

**Table 1:** Histopathologic features and viral spread in nasal turbinates, tracheae, and lungs of pigs.

	Nasal turbinates		Tracheae		Lungs	
	H&E	IHC	H&E	IHC	H&E^#^	IHC
Control	0/3	−	0/3	−	0/9	−
		−		−		−
		−		−		−
WT	3/3	+++	0/3	−	0/9	−/+
		+++		++		++
		+++		++		+++
R106K	1/3	++	0/3	−/+	2/9	++
		+++		+		+++
		+++		++		+++
Y17H	1/3	−	0/3	−	0/9	−
		−		−		−
		+++		++		+++

Pigs were inoculated as in Fig 2. TBAL fluid and tissues were collected on day 3 after inoculation and prepared as explained in the *Material and Methods* section. Pieces of fixed tissue were processed for histologic analysis, subjected to immunohistochemical staining with influenza NP-specific antibody (IHC) or stained with hematoxylin and eosin (H&E), and observed by microscopy in a blinded manner. The extent of NP staining in tissues is reported as follows: −, negative; −/+, rare positive cells; +, a few positive cells; ++, many positive cells; +++, most cells positive. The number of animals showing signs of pathology by H&E is reported.

^#^Three lobes (right cranial, middle, and caudal) were prepared for each animal (n = 3; 9 lobes in total) and observed separately. Representative pictures are shown in S2 Fig.

All 3 viruses were mildly pathogenic, which is consistent with other studies on pH1N1 infection in pigs [55,57]. The animals were monitored daily for the following signs, which were not observed in any of the pigs: biting, aggression, squealing, increased scent marking, restless/constant walking and slipping, self-mutilation, diarrhea, weight loss, and open-mouthed breathing/gasping. For pigs infected with any of the 3 viruses, we found no notable lesions in the lungs or trachea, except in 1 animal infected with R106K (Table 1 and S2 Fig). This pig had attenuated epithelium (damaged and lost columnar epithelium that was replaced by a thin flattened epithelium covering the basement membrane) in some bronchioles as well as cell debris in some alveoli and bronchioles. It is possible that some lesions may not have been included in the analyses because of the large sizes of the tissues. All 3 pigs inoculated with WT virus had damage to their nasal turbinates, characterized by multifocal ulcerated areas containing granulocytic inflammation. In contrast, similar observations were made in only a third of pigs infected with R106K or Y17H. Compared to the Y17H- or PBS-inoculated groups, WT- and R106K-infected pigs had more infiltrating cells in their TBAL fluid (S3A Fig), as well as increased levels of mRNAs encoding proinflammatory cytokines (i.e., IL-1β and IL-6) and chemokines (i.e., MIP2α and MCP1) (S3B Fig). Cellular infiltration and transcription of inflammatory genes in the lungs of pigs inoculated with Y17H virus were minimal. Overall, the HA-stabilizing mutation R106K (pH 5.3) supported pH1N1 growth, spread, and pathogenicity comparable to those observed with WT virus (pH 5.5), whereas the HA-destabilizing Y17H mutation (pH 6.0) resulted in delayed or reduced virus growth, spread, and pathogenicity.

### Swine support the transmission of pH1N1 viruses with broad HA stability to both swine and ferrets

**Fig 3:**
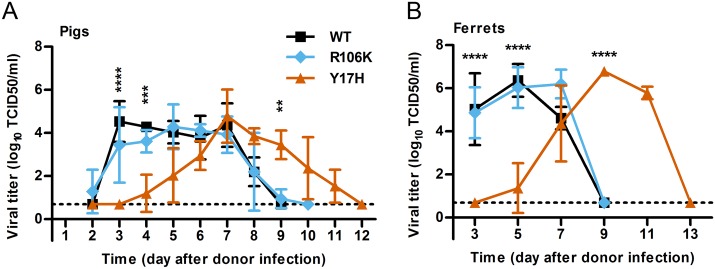
Viral growth in contact pigs and ferrets during transmission. Transmission experiments were performed as described in Table 2. Mean (± SD) viral titers in the nasal cavities of pigs (A) (n = 3) and ferrets (B) (n = 3) were determined by TCID50 titration. Two-way ANOVA with Bonferroni multiple comparisons was performed. Significant differences between the WT and Y17H groups are indicated as follows: **P < 0.01, ***P < 0.001, ****P < 0.0001. There was no significant difference between the WT and R106K groups.

To study swine-to-swine and swine-to-ferret transmission, we co-housed naïve pigs (the contact pig group) in the same pen as donor pigs and positioned cages of naïve ferrets approximately 30 cm from the pigpen (the ferret group). We collected nasal swabs from swine daily and nasal washes from ferrets every other day. There was 100% transmission of all 3 viruses (3/3) in both the contact pig and ferret groups (Table 2), as assessed by positive viral titers from piglet nasal swabs and ferret nasal washes (Fig 3) and by serum antibody titers 2 weeks post-infection (S1 Fig). The average peak titers were similar for all 3 viruses in both host species (Fig 3). For WT virus transmitted by contact transmission to pigs and by airborne transmission to ferrets, the average times of first detection and peak infection were approximately 3 and 4 days after donors were infected, respectively (Table 2). The transmission timing of the stabilized R106K virus was similar to that of WT virus (P > 0.2). In contrast, the transmission of the destabilized Y17H virus was significantly delayed (P < 0.04), with the first detection and peak of infection occurring at averages of 5.0 and 7.3 days after donor inoculation, respectively, in pigs and 6.3 and 9.0 days after donor inoculation, respectively, in ferrets (Table 2).

**Table 2:** Detection of transmission in contact pigs and ferrets.

		Transmission	First day of detection	*P (t*-test)	Peak day of infection	*P (t*-test)
Pig	WT	3/3	3.0		4.0 (± 1.4)	
	R106K	3/3	2.7 (± 0.5)	0.374	5.3 (± 1.7)	0.442
	Y17H	3/3	5.0 (± 0.8)	0.026	7.3 (± 0.5)	0.034
Ferret	WT	3/3	3.0		4.3 (± 0.9)	
	R106K	3/3	3.0		5.7 (± 0.9)	0.230
	Y17H	3/3	6.3 (± 0.9)	0.007	9.0	0.002

Donor pigs were infected with 1.4 × 10^6^ PFU of WT, Y17H, or R106K virus in PBS. The next day, contact pigs (n = 3) and ferrets (n = 3) were co-housed with donors. Nasal samples were titrated by TCID50. The mean (± SD) day on which virus was first detected and the mean day on which the infection peaked (corresponding to the highest titer) is reported. Time values are reported with the starting time of day 0 as the day that donor pigs were inoculated. Student *t*-tests between mutant viruses and WT were performed: *P*-values and significance (asterisks) are shown.

### HA stability phenotypes and genotypes associated with swine-to-swine and swine-to-ferret transmission

**Fig 4:**
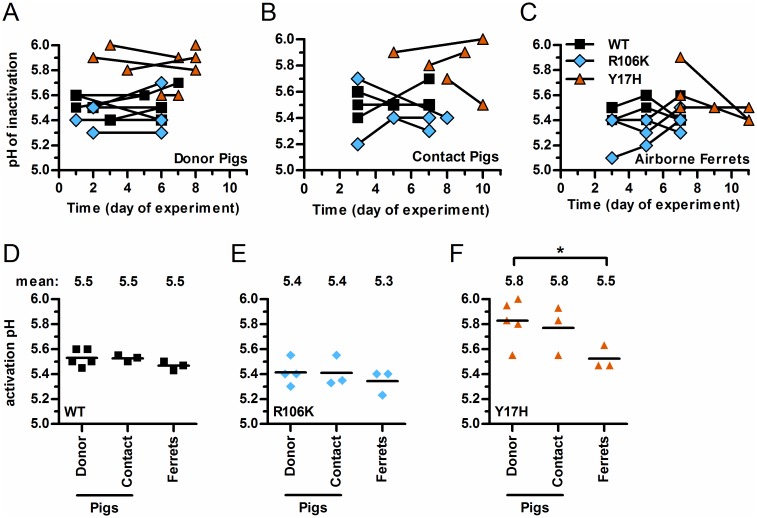
HA acid stability of viruses after inoculation and transmission. Transmission experiments were performed as in Table 2. Viral isolates from nasal swabs and washes of donor pigs, contact pigs, and ferrets were amplified one round in MDCK cells. The HA activation pH of the samples were measured using an acid inactivation assay. The ΔpH values corresponding to the ratio of the pH of the sample to that of the inoculum were used to estimate the activation pH. The means (± SD) of absolute pH values are displayed over time (A, B, and C) and by virus group (D, E, and F). One-way ANOVA analysis followed by a Tukey post-hoc test was performed to compare groups, and significance is indicated as follows: *P < 0.05, **P < 0.01.

To determine if HA stability changed in animals, we measured the HA activation pH of isolates from pig nasal swabs and ferret nasal washes. In the WT-infected/exposed groups of pigs and ferrets, the HA activation pH averaged 5.5 (the input value) and ranged from 5.45 to 5.60 (Fig 4A and 4D). In the R106K-infected/exposed groups, the HA activation pH averaged 5.4 (range, 5.30–5.55) in pigs and 5.3 (range, 5.20–5.40) in ferrets (Fig 4B and 4E). Thus, HA stability phenotypes were maintained in pigs and ferrets infected with WT and R106K viruses. In the Y17H groups, the HA activation pH ranged from 5.55 to 6.00 in donor pigs (average, 5.83), from 5.55 to 5.93 in contact pigs (mean, 5.77), and from 5.47 to 5.63 in ferrets (mean, 5.52) (Fig 4C and 4F). As the input HA activation pH for Y17H was 6.0, average decreases of 0.2 and 0.5 pH units were associated with adaptation and transmission in swine and ferrets, respectively. For swine-to-ferret transmission of Y17H, the measured HA activation pH values for ferret recipients were initially 5.5, 5.6, and 5.9 (Fig 4C). Thus, 2 of the 3 transmission events to ferrets were associated with HA stabilization at 5.5 to 5.6, a stability of pH1N1 that is associated with airborne transmissibility in ferrets and human pandemic potential [39]. In the third ferret, a relatively unstable virus (pH 5.9) was transmitted from a pig to the ferret before becoming stabilized at pH 5.4 (Fig 4C). It is unknown whether the virus was transmitted by large droplets over a short range or by smaller aerosols capable of traveling longer distances. Space constraints in our animal facility prevented wide (> 1 m) separation of the ferret cages from the pig pens.

**Fig 5:**
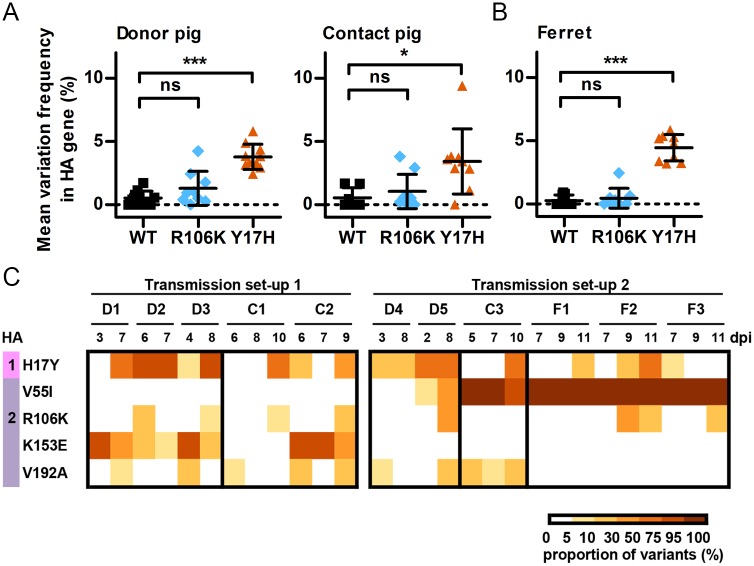
Genotypic analysis of the HA gene in viruses isolated from directly inoculated pigs, from contact pigs, and from ferrets after transmission. Transmission experiments were performed as described in Table 2. Viral RNA was extracted from nasal swabs and washes collected from donor pigs (D1, D2, D3, D4, and D5), contact pigs (C1, C2, and C3) and ferrets (F1, F2, and F3). Gene-specific PCR products were obtained for the HA, NA, and M segments and analyzed by next-generation sequencing (with Illumina MiSeq). (A, B) The mean variation frequency for each sample/time point was calculated by using positions that were variable in at least one of the examined samples/reads and is displayed as the mean pairwise differences between all variants in a sample and the inoculum. One-way ANOVA analysis followed by a Tukey post-hoc test was performed to compare groups, and significant differences are shown (*P<0.05, ***P < 0.001). (C) Heat map of major amino-acid changes occurring in pigs and ferrets in the Y17H group. The results from the 2 independent experiments (transmission set-ups 1 and 2) are shown separately, and only those mutations that were transferred from the donor to a contact pig or ferret are displayed. Variants occurring with a frequency greater than 5% are reported according to H3 numbering and their position in HA1 and HA2. No mutations were found in the NA or M genes. ns, not significant; dpi, day post-inoculation.

From pig nasal swab and ferret nasal wash samples, we sequenced the HA, NA, and M genes, as all 3 genes may alter the HA activation pH [38,58,59]. Consistent with their maintaining an HA activation pH of approximately 5.5, the WT-virus groups showed little nucleotide sequence variation (Fig 5), had only minor populations of HA gene variants, and had no populations of NA or M gene variants (S4 Fig). The activation pH values for the R106K groups in pigs increased by an average of 0.1 pH units and remained at an average of 5.3 in ferrets (Fig 4E). Accordingly, the R106K groups exhibited relatively little sequence variation (Fig 5). Each animal had some populations of one or more minor variants, but these generally did not increase in abundance over time (S5 Fig). No variant with a K253R mutation in the NA protein was found in pigs, whereas the mutation had an abundance of 95% in one R106K-group ferret on day 3, although this decreased to 68% of the virus population within 2 days, suggesting that it is not a preferred mutation. Indeed, it may have limited structural or functional impact on the NA protein, as K253 is a surface residue located on the bottom of the NA head at a turn between two β-strands located distal to both the enzyme active site and the adjacent protomers of the tetramer [60].

**Fig 6:**
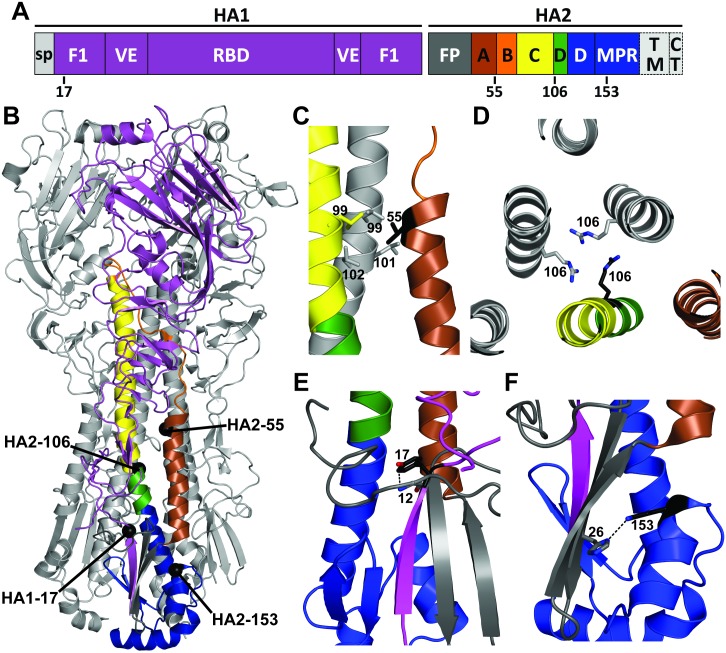
Locations of HA mutations in the prefusion structure. (A) Structural domains in the primary sequence of the H1N1 HA protein. Regions shaded gray are not included in the ectodomain structure. These include the signal peptide (sp), transmembrane domain (TM), and cytoplasmic tail (CT) region. HA1 regions in the ectodomain structure are shaded magenta and include the HA1 chains located in the stalk (F1) and the vestigial esterase (VE) domain and receptor-binding domain (RBD) in the head. HA2 stalk regions include the fusion peptide (FP, gray), helix A (brown), loop B (orange), helix C (yellow), helix D (green and blue), and membrane proximal region (MPR, blue). Color conventions are maintained in panels B-F. (B) Crystal structure of the HA protein of A/CA/04/09 (PDB entry 3UBE [71]) with two protomers shaded in gray. Alpha carbons of highlighted amino acid positions are denoted as black spheres. H3 numbering is used. Residues HA1-17, HA2-V55I, HA2-106, and HA2-K153E are at positions 24, 399, 450, and 497, respectively, after the initiating methionine in the H1N1 HA protein (H1 numbering). HA2-V192A is located in the transmembrane domain and cannot be shown here. (C) Side view of the region surrounding HA2-55, which packs into a pocket of leucine residues at HA2 positions 99, 101, and 102 in two adjacent C helices. (D) Downward view of HA2-106 residues, which are located in the core of the HA2 stalk. (E) Side view of HA1-Y17, which interacts with the HA2-G12 backbone in the fusion peptide. The interatomic distance shown is 3.3 Å. (F) Side view of HA2-K153, which is proximal to HA2-H26. The interatomic distance shown is 3.1 Å.

**Fig 7:**
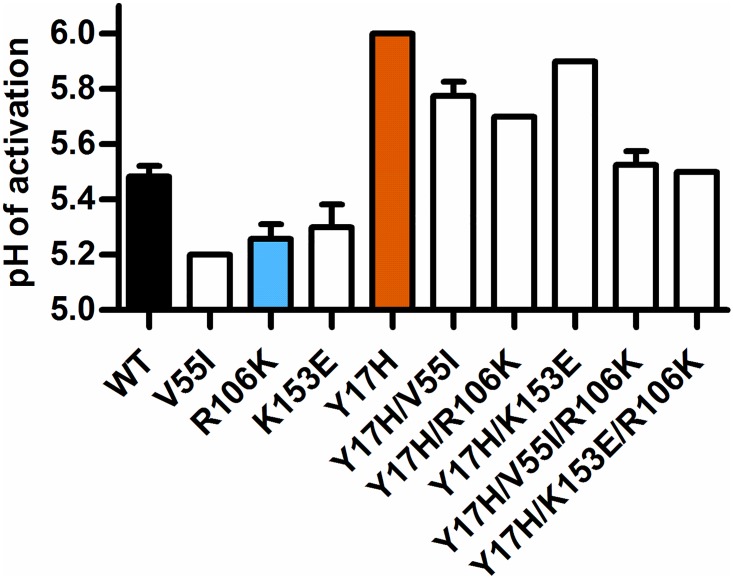
HA activation pH values for recombinant mutant viruses. pH1N1 mutants possessing one or more of the mutations were generated by reverse genetics, and the pH of HA activation was determined using a syncytium assay. The mean (± SD) of the pH values is shown.

The HA sequence variation averaged approximately 2% in the Y17H groups (Fig 5), and subpopulations of viruses containing HA protein mutations emerged (S6 Fig). Minor populations of the NA mutations V46A and K253R were detected in 1 donor pig and 1 contact pig, respectively, but were not associated with transmission. The following five HA mutations located in the stalk region were transmitted to or emerged in contact pigs and/or ferrets: HA1-H17Y (reversion), HA2-V55I, HA2-R106K, HA2-K153E, and HA2-V192A (Fig 6). In both experiments, a proportion of the HA1-H17Y reversion mutation was detected in pigs (donor and contact) and ferrets. HA1-Y17 forms a hydrogen bond with the fusion peptide backbone (Fig 6E), stabilizing the HA protein by approximately 0.5 pH units (Fig 7). In experiment 1, HA2-K153E emerged in each of the 3 donor pigs and in 1 of the contact pigs (S6 Fig). The HA2 residue K153 is located in helix G of the membrane-proximal region. The K153 sidechain may exert electrostatic repulsion with HA2-H26, which is located in the center of one of the two β-strands attached to the fusion peptide (Fig 6F). We generated reverse-genetics viruses containing mutations associated with transmission in the Y17H groups. A K153E mutation reduced the HA activation pH by approximately 0.2 units on the backgrounds of WT pH1N1, Y17H, and Y17H/R106K (Fig 7). In experiment 2, HA2-V55I was a minor population in 1 of the 2 donor pigs, was more than 90% abundant in the contact pig, and reached more than 99% abundance in each of the 3 ferrets (S6 Fig). The HA2 residue V55 is located in helix A, which buttresses the central coiled-coil (Fig 6C). The packing of residue 55 into its pocket may be stabilized by a V55I mutation, which reduced the HA activation pH by approximately 0.2 to 0.3 units (Fig 7). Some isolates contained HA2-V192A substitutions, but their proportions remained below 30%, suggesting that these substitutions did not improve fitness. Minor populations of HA2-R106K were also detected in both experiments. The R106K mutation stabilizes the HA protein by 0.2 to 0.3 pH units, perhaps by reducing electrostatic repulsions at the core of the central triple-stranded coiled-coil at the hinge between helices C and D (Figs 6D and 7).

Overall, mutations associated with Y17H adaptation in pigs and ferrets were found to stabilize the HA protein, consistent with the average HA activation pH for this group shifting from 6.0 to 5.8 in pigs and 5.5 in ferrets (Fig 4). In contrast, the R106K virus, which had an HA stabilized at pH 5.3, retained a stable genotype and phenotype, along with wild-type–like replication and transmissibility in pigs and ferrets by contact and airborne routes, respectively.

## Discussion

Pigs appear to be well suited as an intermediate host within which zoonotic influenza genes may undergo initial mammalian adaptation in advance of a human pandemic. Humanizing adaptations may arise after the reassortment of human and animal influenza viruses, as was the case in the 3 previous pandemics in 1957, 1968, and 2009 [61]. A pandemic virus may also arise after direct adaptation of a swine, avian, or other zoonotic virus in humans. Pigs tolerate the 2 previously known humanizing adaptations required for pandemic potential: a switch in receptor-binding specificity from α-2,3- to α-2,6-linked sialic acid and a decrease in the temperature required for optimal polymerase activity from approximately 41°C to 33°C [54,55]. A third molecular adaptation has recently been discovered to be necessary for airborne transmissibility of H5 influenza viruses in ferrets and pH1N1 pandemic potential in humans: stabilization of the HA protein [36,39,62]. The observed HA activation pH values of H1 and H5 viruses in avian hosts typically, but not always, range from pH 5.5 to 6.0, whereas those of human-adapted H1, H2, and H3 viruses range from approximately 5.1 to 5.6 [33,35,42,63]. Thus, the HA proteins of avian influenza viruses generally appear to be less stable than those of human influenza viruses. We measured the HA activation pH values of circulating H1 and H3 swine influenza viruses and found them to range from 5.1 to 5.9. We also investigated the capacities of engineered pH1N1 viruses with HA activation pH values of 5.3, 5.5, and 6.0 to replicate in pigs and be transmitted from pigs to pigs by contact and from pigs to ferrets by the airborne route. All 3 viruses replicated in 100% of the inoculated pigs and were transmitted with 100% efficiency to contact pigs and ferrets. After transmission, only the input virus with an HA activation pH of 6.0 was genetically unstable, resulting in average HA activation pH values after transmission that ranged from 5.3 to 5.9 in pigs and from 5.2 to 5.6 in ferrets. When these results were combined with surveillance data, pigs were found to support a broad range of HA activation pH (5.1–5.9), consistent with the notion that this species serves as a bridging host for HA stabilization, similar to its role with respect to receptor binding and polymerase activity.

The cohousing of pigs, both in this experiment and in commercial facilities, may also contribute to a broad range of tolerable HA activation pH for virus transmission. Cohousing of animals allows contact transmission, which was previously shown to be more permissive than airborne transmission between ferrets for the destabilized Y17H mutant [39]. Comparing the two experiments, swine in the Y17H group were able to transmit with 100% efficiency to ferrets by the airborne route, whereas ferret donors promoted less efficient airborne transmission to ferrets (25%).

Given that human influenza A viruses prefer a stable HA protein but many zoonotic viruses have an HA protein that is relatively unstable [35,39], it is important to identify the evolutionary pathways by which an unstable HA could acquire increased stability. The 2009 pH1N1 pandemic virus most likely evolved by a stepwise stabilization pathway, as HA acid stability progressively increased during the evolution of H1N1 from swine precursors (pH 5.5–6.0) and early 2009 human isolates (pH 5.5) to later human isolates (pH 5.2–5.4) [35,39,45,64,65]. The surveillance results reported here show that post-2009 swine H1 viruses have a broad range of HA activation pH (5.1–5.9), and experimental infections in pigs show that a pH1N1 virus with an HA activation pH of 5.3 is readily transmitted between swine and from swine to ferrets without attenuation, fixed mutations, or a change in its HA acid stability. Thus, HA stabilization may also occur by a wide-range crossover pathway in which the HA protein becomes stabilized in swine without becoming attenuated. In a recent study, we found that direct inoculation of a pH1N1 virus with an unstable HA protein (pH 6.0) into ferrets can result in HA stabilization within the ferret host [39]. If these results can be extended to humans, then a third pathway to HA stabilization is direct adaptation in humans. HA stabilization was required for the direct adaptation of airborne transmissibility in ferrets by avian H5 viruses containing mutations conferring α-2,6 receptor–binding specificity [36,37,62], further demonstrating the importance of this molecular property in interspecies adaptation. Similarly, the 1957 and 1968 pandemic viruses that emerged after reassortment had stable HA proteins (with activation pH values of approximately 5.1) [35]; however, the HA activation pH values of the prepandemic precursor viruses are unknown.

For pre-2009 H1N1 swine IAVs, the ranges of observed HA activation pH values from classical, Eurasian avian-like, and triple-reassortant lineages were measured as 5.5 to 5.8, 5.5 to 6.0, and 5.4 to 5.7, respectively [39,42]. Thus, the overall observed range is intermediate to unstable at pH 5.4 to 6.0. Here, we measured the HA activation pH values of swine IAVs isolated in 2009 or later. Postpandemic H1N2 viruses (pH 5.5–5.9) had HA acid stability similar to that of prepandemic swine IAVs, whereas postpandemic H1N1 (pH 5.1–5.7) and H3N2 (pH 5.3–5.8) were shifted to lower values that overlap at the bottoms of their ranges with those viruses associated with adaptation to humans [39,45,64,65]. With respect to HA activation pH, contemporary swine H1N1 and H3N2 IAVs most likely pose a greater pandemic risk than do pre-2009 swine viruses, as isolates of these strains that contain stable (human-like) HA proteins appear to retain fitness. However, it should be noted that pandemic potential is also determined by the antigenic distance of a given zoonotic virus from those that have circulated in humans.

The present findings should heighten appreciation of the threat posed by swine IAVs in terms of causing a future pandemic in humans. Evidence is growing that HA stabilization plays a key role in interspecies adaptation and human pandemic potential [35–37,39,43,44], and here we found that contemporary swine IAVs with a stable HA protein remain fit in swine. Swine also support the other 2 known molecular properties associated with human pandemic potential, namely α-2,6-receptor binding specificity and efficient polymerase activity at 33°C [55]. Given the historical importance of swine IAVs in the emergence of pandemic influenza, surveillance of circulating swine IAVs should be intensified and should include measuring molecular properties associated with human pandemic potential, in addition to gene sequencing and monitoring potential drift away from cross-reactivity with human IAV antibody responses. Close monitoring of IAVs circulating in swine production systems would not only enable further analysis of the natural evolution of swine IAV strains but also help us anticipate the emergence of viruses with pandemic potential, enabling enhanced preparation for and prevention of swine-to-human transmission.

## Materials and methods

### Cell lines and viruses

Madin-Darby Canine Kidney (MDCK), African green monkey kidney (Vero), and human embryonic kidney (HEK 293T) cells were obtained from the American Type Culture Collection. MDCK and Vero cells were maintained in Dulbecco’s modified Eagle’s medium (MEM) supplemented with 5% fetal bovine serum (FBS) and 1% penicillin-streptomycin at 37°C in 5% CO_2_. 293T cells were maintained in Opti-MEM containing 10% FBS at 37°C in 5% CO_2_.

A/Tennessee/1-560/2009 recombinant viruses were generated by reverse genetics with pHW2000 plasmids, each containing an individual gene, being transfected into co-cultures of MDCK and 293T cells as described previously [66]. Amino-acid changes were introduced into the pHW2000-HA plasmid by using the QuikChange site-directed mutagenesis kit (Stratagene, Cedar Creek, TX) in accordance with the manufacturer's instructions. The Y17H and R106K mutants were previously described [39]. Virus stocks were prepared in MDCK cells and titrated by plaque assay. Virus identity and the absence of unintended mutations were confirmed by Sanger sequencing and next-generation sequencing as shown previously [39].

The contemporary swine H1 and H3 influenza viruses described in S1 and S2 Tables were obtained from the repository at St. Jude Children’s Research Hospital (St. Jude) and propagated in MDCK cells.

### Ethics statement

Animal experiments were conducted in an ABSL2+ facility in compliance with the NIH and the Animal Welfare Act and with the approval of the St. Jude Animal Care and Use Committee, protocol number 464.

### Animal experiments

Food and water were provided *ad libitum* to all animals. Animals were observed daily for signs of diseases or stress. The 3-week-old piglets (Midwest Research Swine, Glencoe, MN) and 5-month-old male ferrets (Triple F farms, Sayre, PA) tested negative for IAVs.

Two animal experiments were performed. In both experiments, 1.4 × 10^6^ PFU of virus in PBS was intranasally inoculated using a spray bottle. In experiment 1, we inoculated 3 donor pigs and 1 day later co-housed 2 naïve contact pigs in the pen. Nasal swabs were collected daily for 11 days for viral titer determination, and serum was collected on day 15 of the experiment for seroconversion testing. In experiment 2, we inoculated 5 pigs and 1 day later introduced 1 naive contact pig into the pen and positioned 3 naïve ferrets in cages approximately 30 cm from the pen, thereby allowing the exchange of droplets and aerosol particles. On day 3 of the experiment, 48 h after naïve animals were added, 3 of the directly inoculated pigs were euthanized by intracardiac administration of Euthasol solution (sodium pentobarbital and sodium phenytoin) under anesthesia and exsanguinated. The lungs and trachea were washed with 50 mL PBS containing 2 mM EDTA, and the BALF was harvested for virus titration and cell counting. Nasal turbinates, tracheae, and lungs were collected then homogenized in PBS in the Qiagen Tissue Lyser II, and a TCID50 titration was performed in MDCK cells. Tissues were also used for analyses of cytokine and chemokine mRNA expression and for histopathologic analysis.

### Histologic and immunohistochemical analyses

Swine respiratory samples were collected 3 days after infection. Whole lungs, tracheae, and nasal turbinates were fixed in 10% neutral-buffered formalin, embedded in paraffin, and sectioned. Sections on slides were stained with hematoxylin and eosin or with polyclonal anti–influenza NP antibody and examined by light microscopy in a blinded fashion by a pathologist according to common guidelines.

### Cytokine mRNA analyses

RNAlater-preserved swine tissues were homogenized, and total RNA was extracted using the RNeasy Mini Kit (Qiagen, Germantown, MD). The levels of IFN-α, IL-1β, IL-6, IL-8, MIP2α, and MCP1 mRNA were analyzed by semiquantitative real-time PCR analysis on a 7500 Fast Real-Time PCR system (Applied Biosystems, Waltham, MA). Briefly, mRNA was reverse transcribed using oligo-dT primers and the SuperScript III First-Strand Synthesis System (Invitrogen, Carlsbad, CA). The resulting cDNA was analyzed with specific primers and the QuantiTect SYBR green PCR master mix (Qiagen) in accordance with the manufacturer’s instructions. The primers for swine housekeeping (18S) and cytokine genes were described previously [67]. Samples were analyzed in triplicate. After normalization to 18S, the fold-change ratio of expression in virus-infected to that in control samples was calculated for each gene by using the ΔΔCt method and expressed as 2^−ΔΔCt^.

### Serologic testing

Seroconversion was tested 2 weeks after virus inoculation or contact. Serum was treated with receptor-destroying enzyme (Denka Seiken, Campbell, CA) overnight at 37°C to destroy nonspecific inhibitors, heat-inactivated at 56°C for 30 min, and tested by a hemagglutination inhibition (HI) assay with A/Tennessee/1-560/2009 WT virus and 0.5% turkey red blood cells (Rockland Immunochemicals Inc., Limerick, PA). The HI titer was determined as the reciprocal of the highest serum dilution that completely inhibited hemagglutination.

### Next-generation sequencing

Deep amplicon sequencing was used to determine non-synonymous variations in the HA, NA, and M genes of viruses isolated from unpassaged pig nasal swab and ferret nasal wash samples. Two-step reverse transcription–PCR (RT-PCR), DNA library preparation, and genomic sequence analysis were performed as previously described [39,68]. Briefly, viral RNA was extracted using TRIzol (Ambion, Carlsbad, CA), and cDNA was synthesized via reverse transcriptase PCR with the SuperScript III First-Strand Synthesis System (Invitrogen). Influenza A virus HA, NA, and M gene segments were separately amplified using Phusion High-Fidelity PCR Master Mix with HF Buffer (New England BioLabs, Ipswich, MA) and specific primers. PCR amplicons were purified with the QIAquick Gel Extraction Kit (Qiagen) and prepared using the Nextera XT cDNA Library Prep Kit (Illumina, San Diego, CA) in accordance with the manufacturer’s protocol. High-throughput paired-end sequencing was performed using a 2 × 150-bp cycle on an Illumina MiSeq platform. Data analysis was performed using CLC Genomics Workbench 8 software (CLC Bio, Aarhus, Denmark) and a custom Quality-Based Variant Detection pipeline. The variants were called if they met the predefined quality scores and were present in both forward and reverse reads at equal ratios. In addition, the minimum variant read frequency was set at 5%, and variants had to be supported by a minimum of 10 reads. All segments sequenced were completely and equally covered. Heat maps were assembled using Excel (Microsoft Office Professional Plus 2010). The mean variation frequency for each sample/time point was calculated by using positions that are variable in at least one of the examined samples/reads. If a site had a non-synonymous mutation with a frequency greater than 5% in any of the samples, the proportion of the variant was included in the numerator; the denominator was the number of variable sites compared to the inoculum.

### HA acid stability

HA activation pH values were determined by syncytium and acid inactivation assays [69,70]. For the syncytium assay, Vero cells were infected with the recombinant viruses or nasal swab/wash samples and appropriate control viruses for 1 h. At 18 to 24 h after infection, HA-expressing cells were treated with 5 μg/mL l-tosylamido-2-phenylmethyl chloromethyl ketone (TPCK)-treated trypsin (Worthington Biochemical, Lakewood, NJ) for 15 min and pH-adjusted PBS buffers for 5 min at 37°C. Cells were incubated in MEM containing 5% FBS for 3 h at 37°C. Cells were fixed and stained with a Protocol Hema 3 kit (Fisher Scientific, Kalamazoo, MI), and syncytium formation was observed by light microscopy. The pH of activation was determined as the highest pH value at which syncytia were observed. To measure the effect of acid exposure on *in vitro* inactivation, 10 μL of nasal swab/wash samples or virus stocks were diluted in 990 μL of pH-adjusted PBS solutions and incubated for 1 h at 37°C. The remaining infectious virus titer was then determined by TCID50 titration. The curves were fitted to an asymmetric (5-parameter) regression model, and the pH50 values were determined as the point at which a 50% change between the maximum and baseline was observed.

### Statistical analyses

Student’s *t*-test, 1-way ANOVA followed by a Tukey post-hoc test, and 2-way ANOVA with the Bonferroni test were used to compare groups. *P*-values of less than 0.05 were considered significant. All statistical analyses were performed with GraphPad Prism 5 software.

## Supporting information

S1 Fig Neutralizing antibody titers in pigs and ferrets. Donor pigs (n = 5) were infected with 1.4 × 10^6^ PFU of WT, Y17H, or R106K viruses in PBS. The next day, contact pigs (A) (n = 3) and ferrets (B) (n = 3) were co-housed with donors. Blood was collected 14 to 15 days after inoculation/contact, and antibody levels were determined by HI assay and are reported as the mean ± SD.
(TIF)

S2 Fig Representative pictures showing histologic and immunohistochemical findings in control, WT, R106K, and Y17H-infected pigs. Tissues from the lungs (A), trachea (B), nasal respiratory (C) and olfactory neuroepithelium (D) were stained with hematoxylin and eosin (H&E) or with an antibody specific to influenza NP (IHC). The lesions include multifocal ulcerations with granulocytic inflammation in the nasal turbinates and the presence of cell debris in the alveoli and bronchial lumen, as well as attenuated epithelium in some bronchioles, as reported in Table 1. 60× magnification.
(TIF)

S3 Fig Inflammation in the lungs of pigs after inoculation. TBAL fluid and tissues were collected on day 3 after inoculation and examined for signs of lung injury, including cell infiltration in the airways and the release of proinflammatory mediators. (A) Mean (± SD) number of infiltrating inflammatory cells in the TBAL fluid. (B) Mean (± SD) fold change in the cytokine and chemokine concentration in lung tissues as determined by real-time RT-PCR. *P < 0.05 by Student’s *t*-test.
(TIF)

S4 Fig Comprehensive genotypic analysis of HA, NA, and M genes after inoculation of WT and transmission. The experimental procedures were as in Fig 5. RNA was extracted from nasal samples of donor pigs (D1, D2, and D3) inoculated with WT virus and of contact pigs (C1, C2, and C3) and ferrets (F1, F2, and F3) after transmission. Heat maps display the frequency of the mutations among the viral population in each group (> 5% for the HA and gene). HA1 and HA2 are H3 numbering. There were no mutations in the NA and M genes. sp, HA signal peptide; del, deletion; dpi, day post-inoculation.
(TIF)

S5 Fig Comprehensive genotypic analysis of HA, NA, and M genes after inoculation of R106K and transmission. The experimental procedures were as in Fig 5. RNA was extracted from nasal samples of donor pigs (D1, D2, and D3) inoculated with R106K virus and of contact pigs (C1, C2, and C3) and ferrets (F1, F2, and F3) after transmission. Heat maps display the frequency of the mutations among the viral population in each group (> 5% for the HA gene, > 30% for the NA gene). HA1 and HA2 are H3 numbering. There was no mutation in the M gene. del, deletion; ins, insertion; dpi, day post-inoculation.
(TIF)

S6 Fig Comprehensive genotypic analysis of HA, NA, and M genes after inoculation of Y17H and transmission. The experimental procedures were as in Fig 5. RNA was extracted from nasal samples of donor pigs (D1, D2, and D3) inoculated with Y17H virus and of contact pigs (C1, C2, and C3) and ferrets (F1, F2, and F3) after transmission. Heat maps display the frequency of the mutations among the viral population in each group (> 5% for the HA gene, > 30% for the NA gene). HA1 and HA2 are H3 numbering. There was no mutation in the M gene. sp, HA signal peptide; del, deletion; ins, insertion; dpi, day post-inoculation.
(TIF)

S1 Table Contemporary swine H1N1 and H1N2 influenza viruses isolated after 2009. H1N1 and H1N2 viruses isolated after 2009 were assayed for HA activation pH. For each virus the subtype and lineage of HA, NA, and M gene is also reported.
(DOCX)

S2 Table Contemporary swine H3N2 influenza viruses isolated after 2010. H3N2 virus isolate names and associated HA activation pH values are described. (DOCX)
