# Supplementary figures and images for: H1N1 influenza viruses varying widely in hemagglutinin stability transmit efficiently from swine to swine and to ferrets
Source: PLoS Pathog. 2017 Mar 10;13(3):e1006276. doi: 10.1371/journal.ppat.1006276 (PMC5362248; doi:10.1371/journal.ppat.1006276)

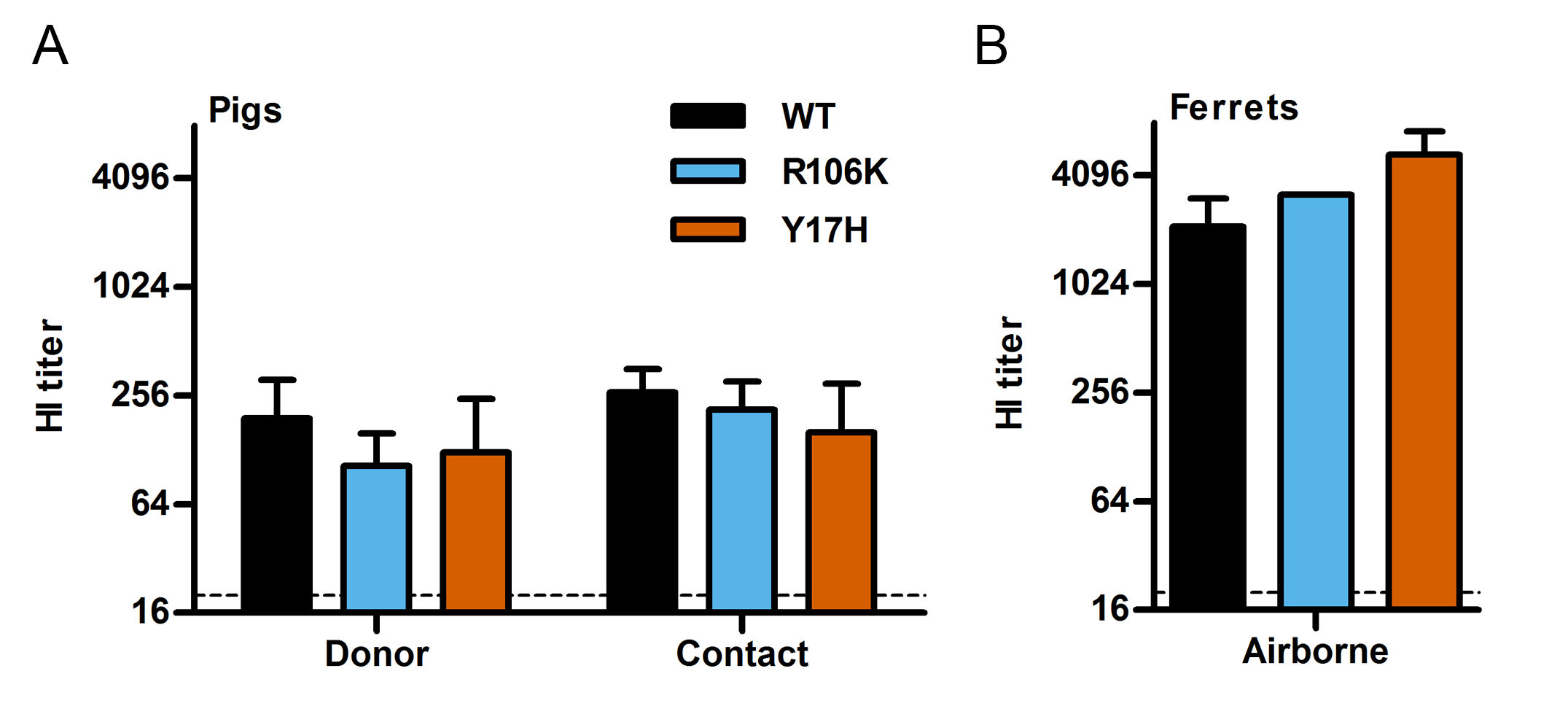

Supplement: S1 Fig — Donor pigs (n = 5) were infected with 1.4 × 10^6^ PFU of WT, Y17H, or R106K viruses in PBS. The next day, contact pigs (A) (n = 3) and ferrets (B) (n = 3) were co-housed with donors. Blood was collected 14 to 15 days after inoculation/contact, and antibody levels were determined by HI assay and are reported as the mean ± SD. (TIF) [file ppat.1006276.s001.tif]

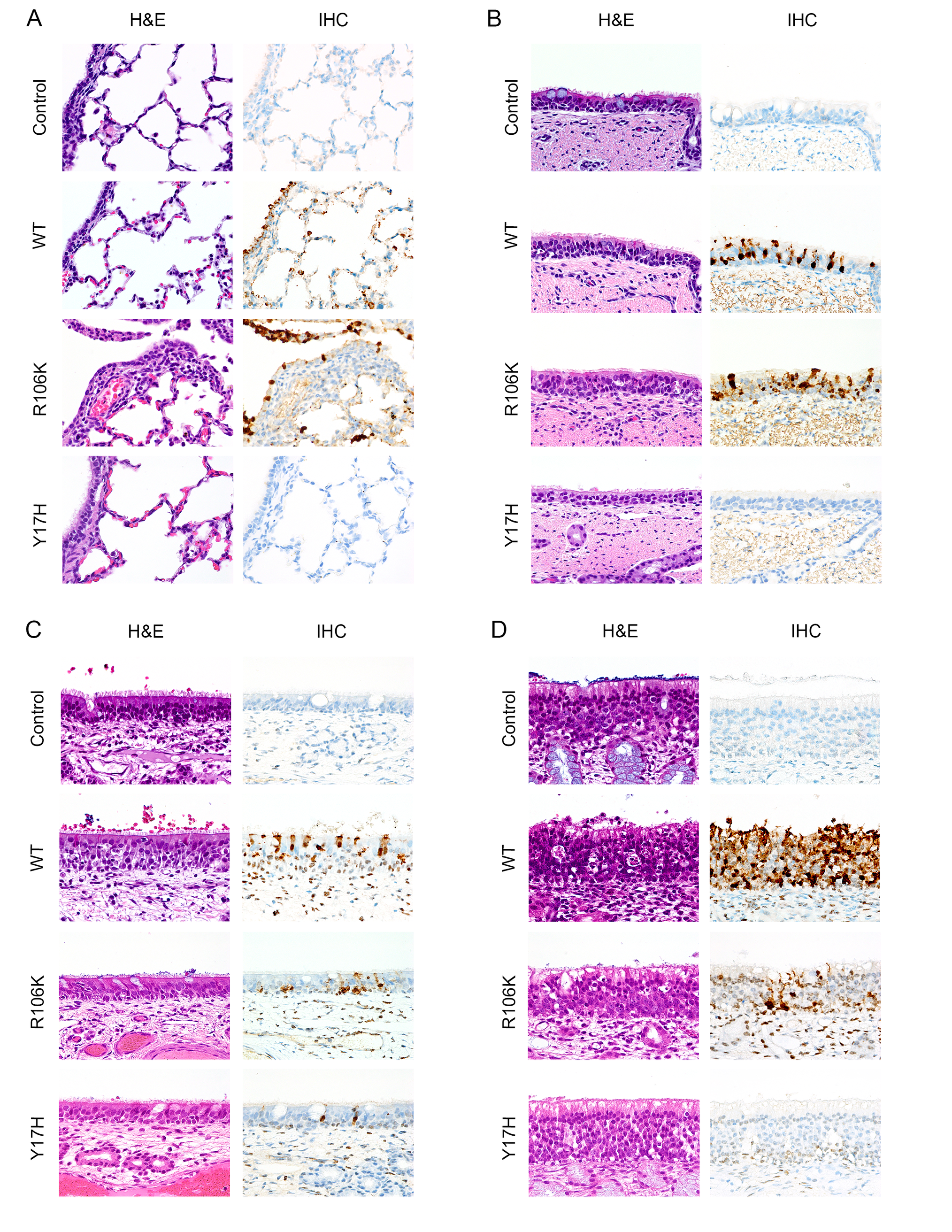

Supplement: S2 Fig — Tissues from the lungs (A), trachea (B), nasal respiratory (C) and olfactory neuroepithelium (D) were stained with hematoxylin and eosin (H&E) or with an antibody specific to influenza NP (IHC). The lesions include multifocal ulcerations with granulocytic inflammation in the nasal turbinates and the presence of cell debris in the alveoli and bronchial lumen, as well as attenuated epithelium in some bronchioles, as reported in Table 1. 60× magnification. (TIF) [file ppat.1006276.s002.tif]

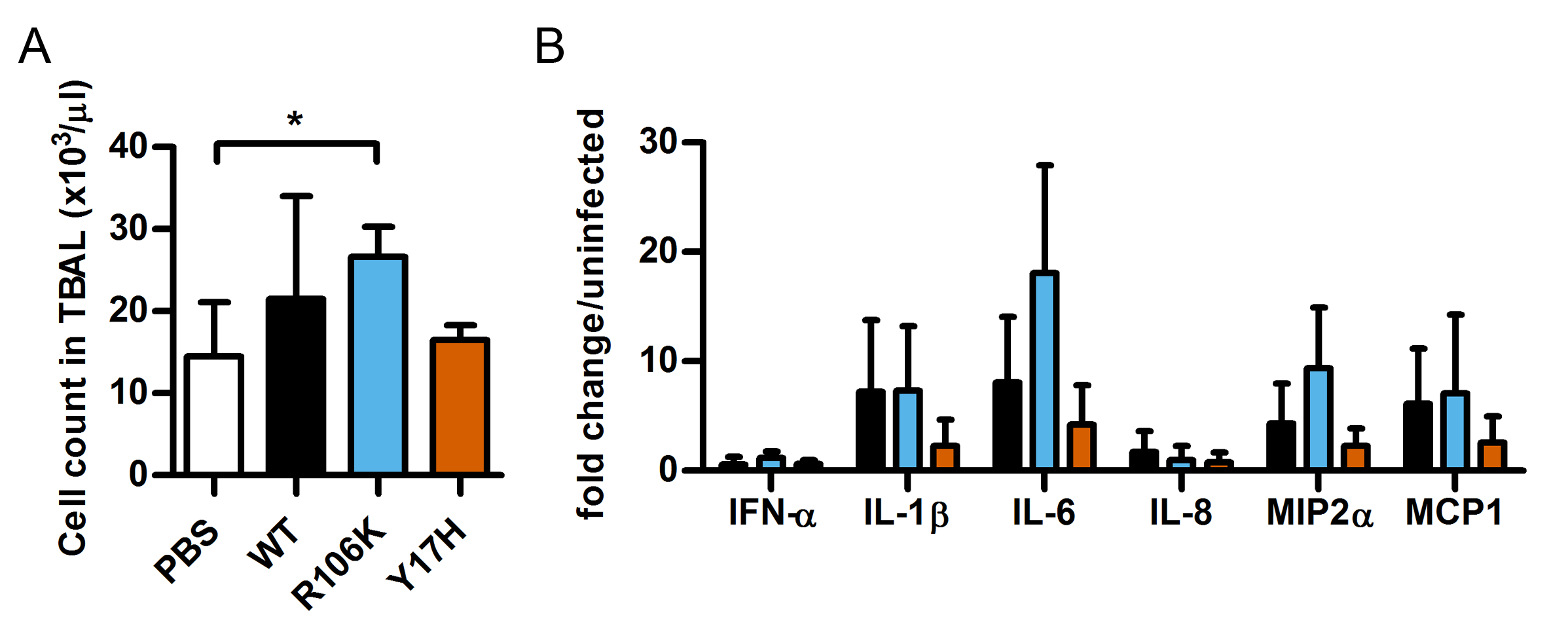

Supplement: S3 Fig — TBAL fluid and tissues were collected on day 3 after inoculation and examined for signs of lung injury, including cell infiltration in the airways and the release of proinflammatory mediators. (A) Mean (± SD) number of infiltrating inflammatory cells in the TBAL fluid. (B) Mean (± SD) fold change in the cytokine and chemokine concentration in lung tissues as determined by real-time RT-PCR. *P < 0.05 by Student’s t-test. (TIF) [file ppat.1006276.s003.tif]

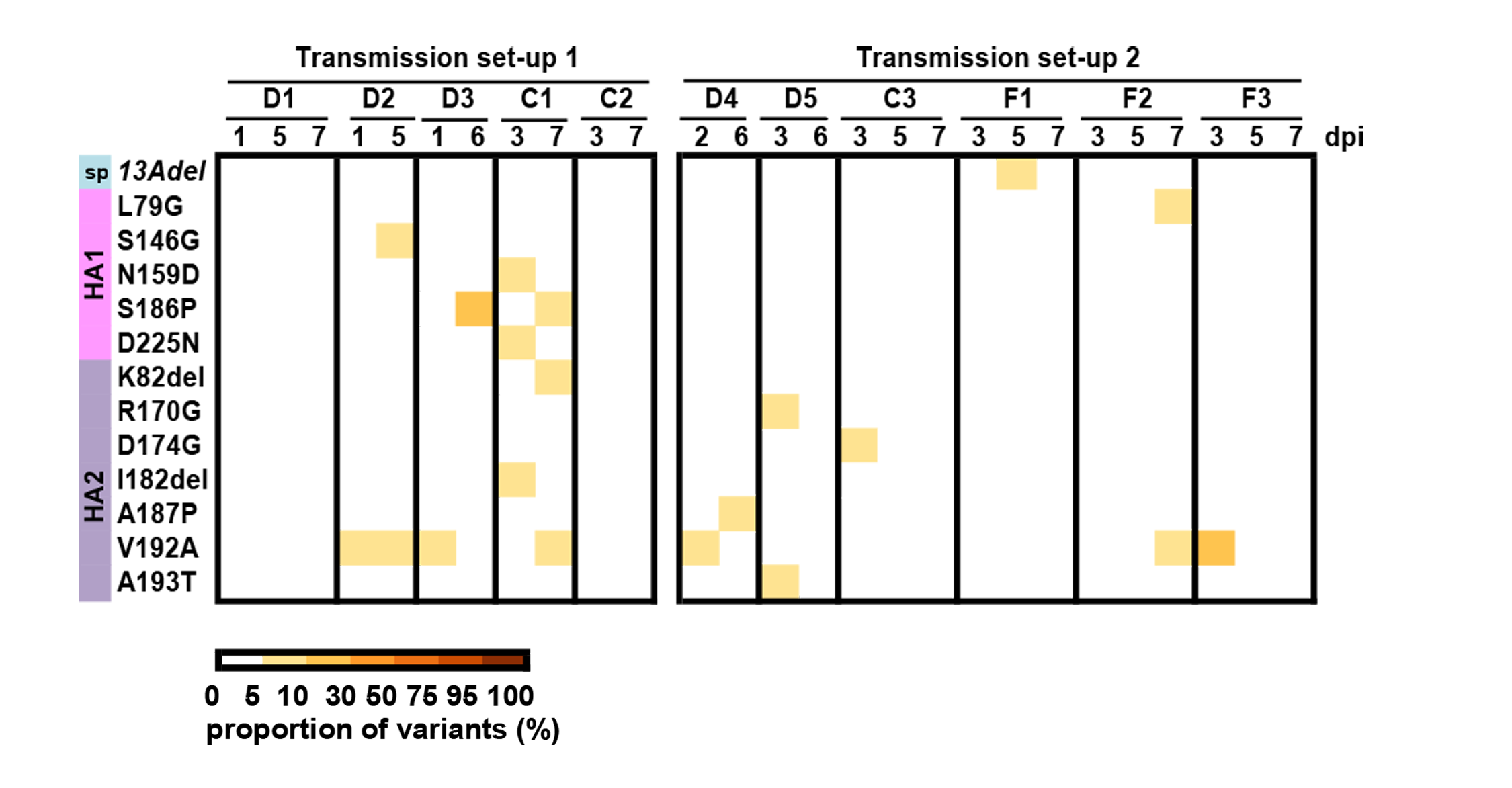

Supplement: S4 Fig — The experimental procedures were as in Fig 5. RNA was extracted from nasal samples of donor pigs (D1, D2, and D3) inoculated with WT virus and of contact pigs (C1, C2, and C3) and ferrets (F1, F2, and F3) after transmission. Heat maps display the frequency of the mutations among the viral population in each group (> 5% for the HA and gene). HA1 and HA2 are H3 numbering. There were no mutations in the NA and M genes. sp, HA signal peptide; del, deletion; dpi, day post-inoculation. (TIF) [file ppat.1006276.s004.tif]

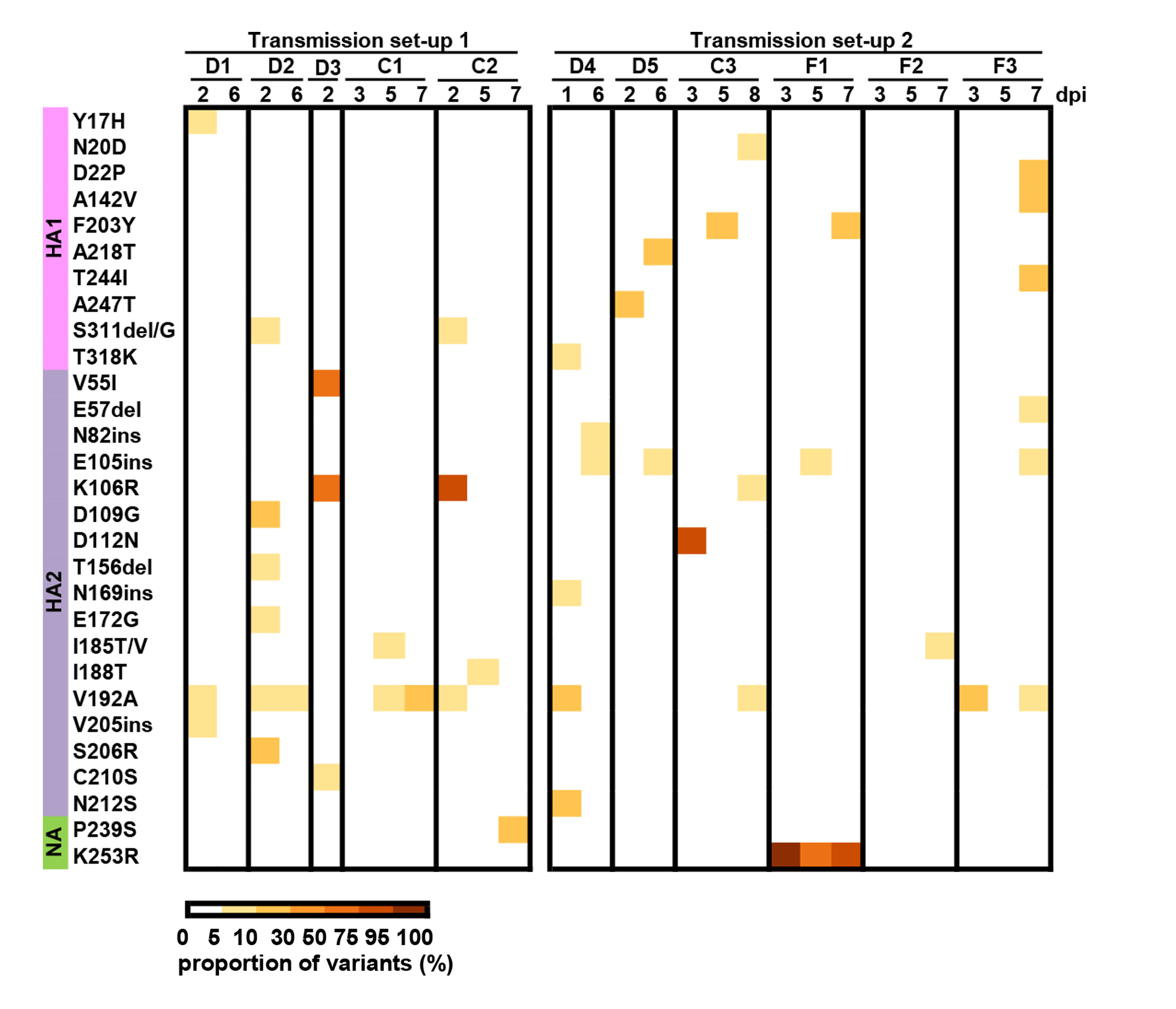

Supplement: S5 Fig — The experimental procedures were as in Fig 5. RNA was extracted from nasal samples of donor pigs (D1, D2, and D3) inoculated with R106K virus and of contact pigs (C1, C2, and C3) and ferrets (F1, F2, and F3) after transmission. Heat maps display the frequency of the mutations among the viral population in each group (> 5% for the HA gene, > 30% for the NA gene). HA1 and HA2 are H3 numbering. There was no mutation in the M gene. del, deletion; ins, insertion; dpi, day post-inoculation. (TIF) [file ppat.1006276.s005.tif]

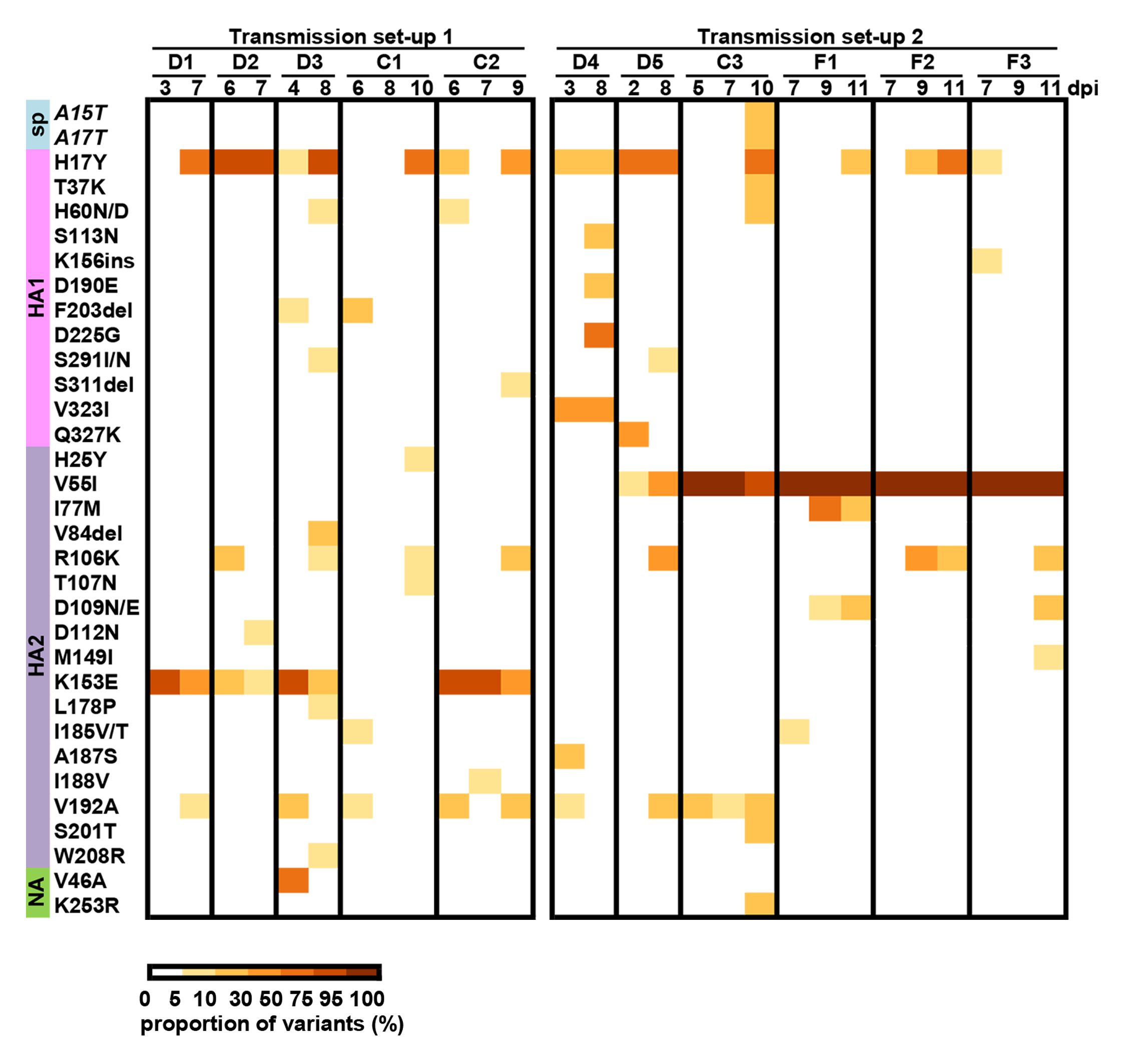

Supplement: S6 Fig — The experimental procedures were as in Fig 5. RNA was extracted from nasal samples of donor pigs (D1, D2, and D3) inoculated with Y17H virus and of contact pigs (C1, C2, and C3) and ferrets (F1, F2, and F3) after transmission. Heat maps display the frequency of the mutations among the viral population in each group (> 5% for the HA gene, > 30% for the NA gene). HA1 and HA2 are H3 numbering. There was no mutation in the M gene. sp, HA signal peptide; del, deletion; ins, insertion; dpi, day post-inoculation. (TIF) [file ppat.1006276.s006.tif]
