# Supplementary material for: H1N1 influenza viruses varying widely in hemagglutinin stability transmit efficiently from swine to swine and to ferrets
Source: PLoS Pathog. 2017 Mar 10;13(3):e1006276. doi: 10.1371/journal.ppat.1006276 (PMC5362248; doi:10.1371/journal.ppat.1006276)
Supplement: S1 Table — For each virus the subtype and lineage of HA, NA, and M gene is also reported. (DOCX) [file ppat.1006276.s007.docx]

**S1 Table. Contemporary swine H1N1 and H1N2 influenza viruses isolated after 2009.**

| Virus | Subtype | HA | NA | M | pH |
| --- | --- | --- | --- | --- | --- |
| TN/1-560/2009 |  | Pandemic | N1 Pandemic | Pandemic | 5.5 |
| sw/TX/13TOSU0049/2013 | H1N2 | δ1 | N2 | Pandemic | 5.9 |
| sw/NE/4D-0114-P14/2014 | H1N2 | Pandemic | N2 | Pandemic | 5.9 |
| sw/IL/2E-1212-P5/2012 | H1N2 | δ1 | N2 | TRIG | 5.8 |
| sw/OH/13TOSU0888/2013 | H1N2 | γ | N2 | Pandemic | 5.8 |
| sw/IL/2G-0113-P16/2013 | H1N1 | γ-pandemic-like | N1 Classical | Pandemic | 5.7 |
| sw/IL/2E-0113-P8/2013 | H1N1 | γ-pandemic-like | N1 Classical | Pandemic | 5.7 |
| sw/IN/28-0715/2011 | H1N1 | γ | N1 Classical | TRIG | 5.6 |
| sw/IL/B1512DTW23/2016 | H1N1 | γ-pandemic-like | N1 Classical | Pandemic | 5.6 |
| sw/IN/28-0818/2011 | H1N2 | δ1 | N2 | Pandemic | 5.6 |
| sw/IL/2F-0113-P30/2013 | H1N1 | γ | N1 Classical | Pandemic | 5.6 |
| sw/OH/09SW1489E/2009 | H1N2 | β | N2 | TRIG | 5.5 |
| sw/NE/4G-0314-P24/2014 | H1N1 | γ | N1 Pandemic | Pandemic | 5.3 |
| sw/GA/1E-0214-P26/2014 | H1N1 | Pandemic | N1 Pandemic | Pandemic | 5.2 |
| sw/NE/4G-0314-P18/2014 | H1N1 | Pandemic | N1 Pandemic | Pandemic | 5.1 |

Swine viruses were isolated during epidemiologic surveys of pig farms. Sequences were obtained through GenBank or by sequencing in-house. The origin of each segment is reported as previously described [[52](#_ENREF_52)]. TRIG, triple-reassortant internal gene.
