# Supplementary material for: H1N1 influenza viruses varying widely in hemagglutinin stability transmit efficiently from swine to swine and to ferrets
Source: PLoS Pathog. 2017 Mar 10;13(3):e1006276. doi: 10.1371/journal.ppat.1006276 (PMC5362248; doi:10.1371/journal.ppat.1006276)
Supplement: S2 Table — (DOCX) [file ppat.1006276.s008.docx]

**S2 Table. Contemporary swine H3N2 influenza viruses isolated after 2010.**

| Virus | Subtype | pH |
| --- | --- | --- |
| sw/MN/3407/2010 | H3N2 | 5.4 |
| sw/MN/3409/2010 | H3N2 | 5.5 |
| sw/OH/3809-1/2010 | H3N2 | 5.6 |
| sw/OH/3809-2/2010 | H3N2 | 5.6 |
| sw/NC/3976/2010 | H3N2 | 5.6 |
| sw/IA/3977/2010 | H3N2 | 5.6 |
| sw/OH/3987/2010 | H3N2 | 5.8 |
| sw/NC/4003/2010 | H3N2 | 5.3 |
| sw/OK/4011/2010 | H3N2 | 5.6 |
| sw/MN/4028/2010 | H3N2 | 5.5 |
| sw/NC/0033/2011 | H3N2 | 5.6 |
| sw/NC/0043/2011 | H3N2 | 5.4 |
| sw/NC/0067/2011 | H3N2 | 5.6 |
| sw/NC/0080/2011 | H3N2 | 5.6 |
| sw/NC/0279/2011 | H3N2 | 5.4 |
| sw/IN/0307/2011 | H3N2 | 5.5 |
| sw/OH/0365/2011 | H3N2 | 5.5 |
| sw/NC/0375/2011 | H3N2 | 5.4 |
| sw/IN/0392/2011 | H3N2 | 5.5 |
| sw/IN/0393/2011 | H3N2 | 5.7 |
| sw/NC/0405/2011 | H3N2 | 5.7 |
| sw/NC/0453/2011 | H3N2 | 5.4 |
| sw/IO/0513/2011 | H3N2 | 5.6 |
| sw/NC/0570/2011 | H3N2 | 5.5 |
| sw/MN /0624/2011 | H3N2 | 5.4 |
| sw/NC/0642/2011 | H3N2 | 5.8 |
| sw/NC/0668/2011 | H3N2 | 5.7 |
| sw/IN/0736/2011 | H3N2 | 5.7 |
| sw/IA/2856/2010 | H3N2 | 5.4 |
| sw/NC/3460/2010 | H3N2 | 5.6 |
| sw/OK/3919/2010 | H3N2 | 5.6 |
| sw/NC/4008-2/2010 | H3N2 | 5.4 |
| sw/MN/4157/2010 | H3N2 | 5.3 |
| sw/TX/0189/2011 | H3N2 | 5.3 |
| sw/AR/0644/2011 | H3N2 | 5.6 |
| sw/MN /0737/2011 | H3N2 | 5.5 |
| sw/AZ/0934/2011 | H3N2 | 5.4 |
| sw/NC/1256/2011 | H3N2 | 5.8 |
| sw/IA/2514-1/2011 | H3N2 | 5.5 |
| sw/IA/2514-2/2011 | H3N2 | 5.6 |
| sw/IA/2514-3/2011 | H3N2 | 5.7 |
| sw/IA/2514-4/2011 | H3N2 | 5.3 |
| sw/KS/2517/2011 | H3N2 | 5.5 |
| sw/OK/2758/2011 | H3N2 | 5.5 |
| sw/MN/2930/2011 | H3N2 | 5.4 |
| sw/MN/3067/2011 | H3N2 | 5.6 |
| sw/TX/3122-1/2011 | H3N2 | 5.7 |
| sw/TX/3122-2/2011 | H3N2 | 5.6 |
| sw/NC/3406/2011 | H3N2 | 5.8 |
| sw/NC/3414/2011 | H3N2 | 5.5 |
| sw/NC/3416/2011 | H3N2 | 5.4 |
| sw/NC/3496/2011 | H3N2 | 5.6 |
| sw/NC/3500/2011 | H3N2 | 5.7 |
| sw/NC/3572/2011 | H3N2 | 5.7 |
| sw/NC/3711/2011 | H3N2 | 5.4 |
| sw/MN/3908-1/2011 | H3N2 | 5.6 |
| sw/MN/3908-2/2011 | H3N2 | 5.7 |

Swine viruses were isolated during epidemiologic surveys of pig farms.
